# Spatial deconvolution from bulk DNA methylation profiles determines intratumoral epigenetic heterogeneity

**DOI:** 10.1186/s13578-024-01337-y

**Published:** 2025-01-23

**Authors:** Binbin Liu, Yumo Xie, Yu Zhang, Guannan Tang, Jinxin Lin, Ze Yuan, Xiaoxia Liu, Xiaolin Wang, Meijin Huang, Yanxin Luo, Huichuan Yu

**Affiliations:** 1 Department of Colorectal Surgery, The Sixth Affiliated Hospital, Sun Yat-sen University, Guangzhou, 510655 Guangdong China; 2 Guangdong Provincial Key Laboratory of Colorectal and Pelvic Floor Diseases, Guangdong Institute of Gastroenterology, The Sixth Affiliated Hospital, Sun Yat-sen University, 26 Yuancun Erheng Road, Guangzhou, 510655 Guangdong China; 3 Guangdong Institute of Gastroenterology, Guangzhou, 510655 Guangdong China; 4 Biomedical Innovation Center, The Sixth Affiliated Hospital, Sun Yat-sen University, Guangzhou, 510655 Guangdong China; 5 Ministry of Education, Key Laboratory of Human Microbiome and Chronic Diseases (Sun Yat-sen University), Guangzhou, Guangdong China; 6 Innovation Center of the Sixth Affiliated Hospital, School of Life Sciences, Sun Yat-sen University, Guangzhou, Guangdong China

**Keywords:** DNA methylation, Intratumor heterogeneity, Epigenetic, Colorectal cancer

## Abstract

**Background:**

Intratumoral heterogeneity emerges from accumulating genetic and epigenetic changes during tumorigenesis, which may contribute to therapeutic failure and drug resistance. However, the lack of a quick and convenient approach to determine the intratumoral epigenetic heterogeneity (eITH) limit the application of eITH in clinical settings. Here, we aimed to develop a tool that can evaluate the eITH using the DNA methylation profiles from bulk tumors.

**Methods:**

Genomic DNA of three laser micro-dissected tumor regions, including digestive tract surface, central bulk, and invasive front, was extracted from formalin-fixed paraffin-embedded sections of colorectal cancer patients. The genome-wide methylation profiles were generated with methylation array. The most variable methylated probes were selected to construct a DNA methylation-based heterogeneity (MeHEG) estimation tool that can deconvolve the proportion of each reference tumor region with the support vector machine model-based method. A PCR-based assay for quantitative analysis of DNA methylation (QASM) was developed to specifically determine the methylation status of each CpG in MeHEG assay at single-base resolution to realize fast evaluation of epigenetic heterogeneity.

**Results:**

In the discovery set with 79 patients, the differentially methylated CpGs among the three tumor regions were found. The 7 most representative CpGs were identified and subsequently selected to develop the MeHEG algorithm. We validated its performance of deconvolution of tumor regions in an independent cohort. In addition, we showed the significant association of MeHEG-based epigenetic heterogeneity with the genomic heterogeneity in mutation and copy number variation in our in-house and TCGA cohorts. Besides, we found that the patients with higher MeHEG score had worse disease-free and overall survival outcomes. Finally, we found dynamic change of epigenetic heterogeneity based on MeHEG score in cancer cells under the treatment of therapeutic drugs.

**Conclusion:**

By developing a 7-loci panel using a machine learning approach combined with the QASM assay for PCR-based application, we present a valuable method for evaluating intratumoral heterogeneity. The MeHEG algorithm offers novel insights into tumor heterogeneity from an epigenetic perspective, potentially enriching current knowledge of tumor complexity and providing a new tool for clinical and research applications in cancer biology.

**Supplementary Information:**

The online version contains supplementary material available at 10.1186/s13578-024-01337-y.

## Introduction

Tumor heterogeneity has long been recognized as a hallmark of cancer and represents an ongoing challenge in the development of personalized cancer medicine [1]. Accumulating cellular genetic and epigenetic aberrations during the tumorigenic process foster intratumor heterogeneity (ITH) in a stochastic manner, which is also influenced by clonal selection and microenvironment niches [2, 3]. Several studies have linked the presence of subclones to poor clinical outcomes or the increased tendency of malignant progression [4, 5]. Subclones might harbor a mutation that can drive drug resistance during therapy despite initial striking tumor regression, resulting in therapeutic failure and ultimate patient demise [6, 7]. Therefore, the nature of ITH, as well as how the clonal structure evolves in response to treatment, should be fully explored.

To date, deep investigation of ITH have been allowed by SNP array [8, 9], RNA-seq [10], whole-exome sequencing [11, 12], whole-genome sequencing [13], targeted deep sequencing [14] and ChIP-seq [15]. Even though single-cell sequencing has emerged as an important tool for clonal deconvolution, it is very costly to implement on the scales needed in the study and usually provides limited sequence coverage [16, 17]. Various methods have been proposed to efficiently infer clonal population structure and its consequences [18–21]. Apart from those ITH algorithms based on genomic variation profile, the ITH model using a methylation profile can also be developed using a mathematical modeling approach [22]. Evaluating the ITH level from epigenetic profile can greatly improve the understanding of ITH and clonal architecture and has great potential as a diagnostic and prognostic tool.

Epigenetic ITH (eITH) can be examined at histone modifications, chromatin conformation and DNA methylation. DNA methylation is a major component of epigenetic modification of the genome and presents substantial intratumoral heterogeneity in multiple cancer [22]. Proof for abnormal gain and loss of DNA methylation in tumors has accumulated tremendously in years, indicating its crucial contribution to neoplastic transformation and plasticity [23–25]. Studies on the evolution of DNA methylation from initial tumors to recurrence found that overall DNA methylation levels were dynamic or exhibited consistent patterns across tumor progression [22]. Methylation changes might be early molecular events, and eITH could increase in adenocarcinoma than its precursors of early stages [26, 27]. Moreover, the eITH might be involved in the host immune response against the tumor since mounting evidence has suggested that the demethylating agent, which reversed the high eITH, could reconstruct the immune surveillance of the tumor [28, 29]. Regarding the phenotypes of tumors, complex methylation of ITH was also related to larger tumor size and increased risk of postsurgical recurrence in patients [30].

Colorectal cancer (CRC) is the third most frequent cancer and the second leading cause of cancer death worldwide [31–33]. CRC is a highly heterogeneous disease that comprises various phenotypes, a plastic condition greatly discouraging therapy outcomes and patient survival. The extent of intratumor diversity in CRC has been revealed through studies on transcriptomic [34, 35], proteomics [36, 37], metabolic status [38, 39] and functional response [40–42]. In particular, the recognition of epigenetic diversity in CRC is well documented and extended to exploring intratumor epigenetic heterogeneity [43–45]. Epigenetic alterations in CRC could occur early and be able to exceed the frequency of genetic abnormalities, thus driving tumor initiation and progression [34, 46]. Unveiling intratumor epigenetic heterogeneity in CRC is clinically significant because it can foster evolutionary adaptation, which results in therapeutic failure. This prevailing concept drives a wide variety of biomarker studies on early diagnosis and prediction for treatment response of CRC [47].

**Fig. 1:**
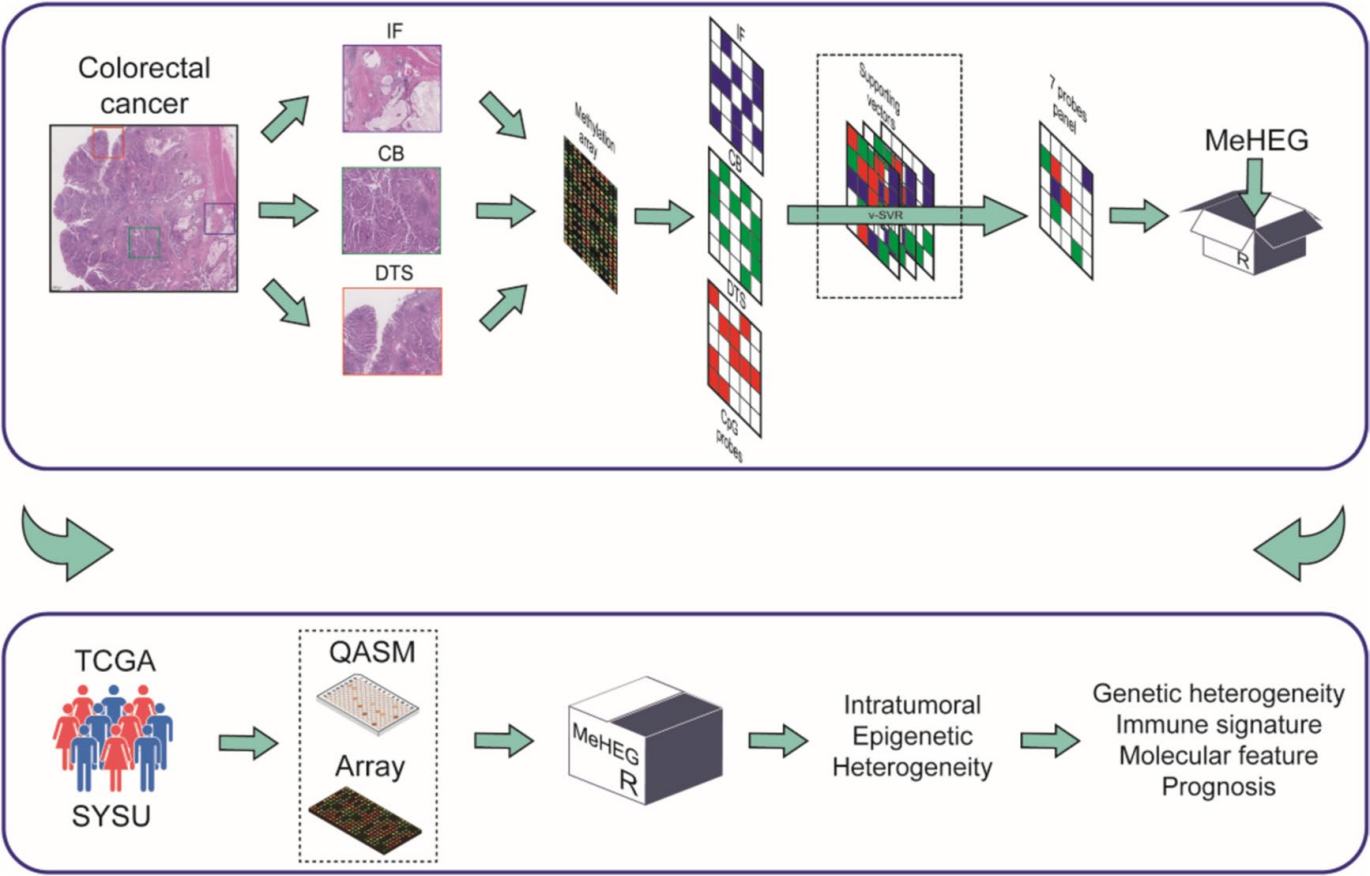
Workflow devised to develop and apply MeHEG tool. *IF* invasive front, *DTS* digestive tract surface, *CB* central bulk, *ν-SVR* nu–support vector regression, *QASM* Quantitative analysis of DNA methylation at single-base resolution

To this end, we applied a deconvolutional method on the differentially methylated probes (DMPs) among digestive tract surface (DTS), central bulk (CB) and invasive front (IF) within individual tumors, and then developed a DNA methylation-based heterogeneity evaluation tool (MeHEG) to facilitate a fast evaluation of eITH (Fig. 1).

## Results

### Development and validation of DNA methylation-based heterogeneity estimation tool

**Fig. 2:**
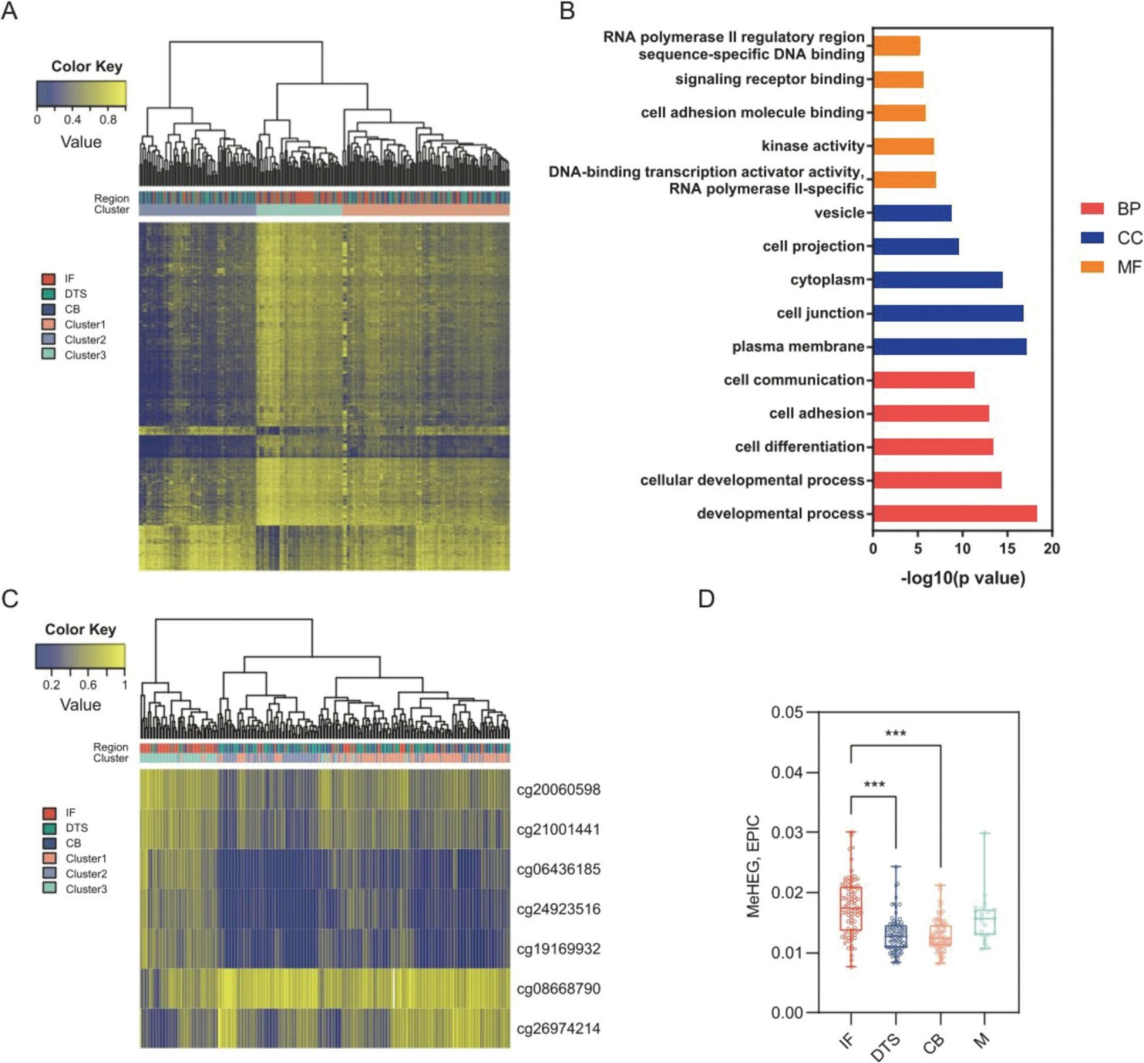
Development and validation of MeHEG. **A** DMPs analysis was performed to identify multiregional methylation profile consisting of 3002 CpG sites with the most statistically significant changes among the three tumor regions (IF, DTS and CB), and the unsupervised hierarchical clustering by these 3002 DMPs yielded 3 clusters. **B** GO analysis was conducted to identify the key pathway including molecular function (MF), biological process (BP) and cell composition (CC). **C** Using these seven probes, obtained by utilizing nu–support vector regression to deconvolve the methylation mixture profile (3002 DMPs), as classifiers, the above yielded three methylation clusters were mapped with unsupervised clustering analysis. **D** The MeHEG scores of the three regions and the metastatic samples (M) of the original study were derived from the 450 k array data. The IF showed a significantly higher MeHEG score than the other two regions. Tukey’s multi-comparison test was applied (***p < 0.001)

First, the tumor region-specific differentially methylated probes (DMPs) were identified from a methylation 450 k BeadChip dataset consisting of 79 CRC patients (HGTP-BHVD cohort). Briefly, the samples of DTS, CB, and IF were resected respectively to construct a 79 × 3 multiregional array and followed by the methylation profiling. A multiregional methylation profile consisting of 3002 CpG sites with the most statistically significant changes among the three tumor regions was identified by DMPs analysis, and the unsupervised hierarchical clustering by these 3002 DMPs yielded 3 clusters, which corresponding to the regional origin (Fig. 2a). From the Gene Ontology (GO) enrichment results, we found that the DMPs were involved in various biological pathways and functions, especially the regulation of tumor initiation, progression, and heterogeneity (Fig. 2b, Supplemental Table S1). The above results suggest that DMPs-based eITH do exist in CRC tissue and is strongly associated with cancer development.

Next, we applied the nu–support vector regression (ν-SVR) to deconvolve the methylation mixture profile (see Methods for details). The final trained model contained seven probes, including cg06436185, cg20060598, cg08668790, cg19169932, cg24923516, cg26974214 and cg21001441 (Supplemental Table S2). Using these probes as classifiers, the above yielded three methylation clusters could be well distinguished by unsupervised clustering analysis (Fig. 2c). Furthermore, by calculating the coefficient of variation of the region- and probe-specific ν-SVR coefficients weighted beta value of the probes, we built a 7-probes based scoring algorithm, named “MeHEG” to infer the eITH of CRC. We have implanted the algorithm into an R package “MeHEG” (available in GitHub) to facilitate calculations. To validate its capacity to reflect the eITH of CRC samples, we calculated the MeHEG score within the three regions. The paired mixed-effect analysis revealed a significant MeHEG score difference among regions (*P* < 0.001) (Fig. 2d), and Tukey’s test showed the IF had the highest MeHEG score than CB and DTS (*P* < 0.001, respectively) (Fig. 2d), which is consistent with the finding of the original research. These results preliminarily confirm the feasibility of MeHEG score-based evaluation for eITH.

### Developing a qPCR-based assay for MeHEG

**Fig. 3:**
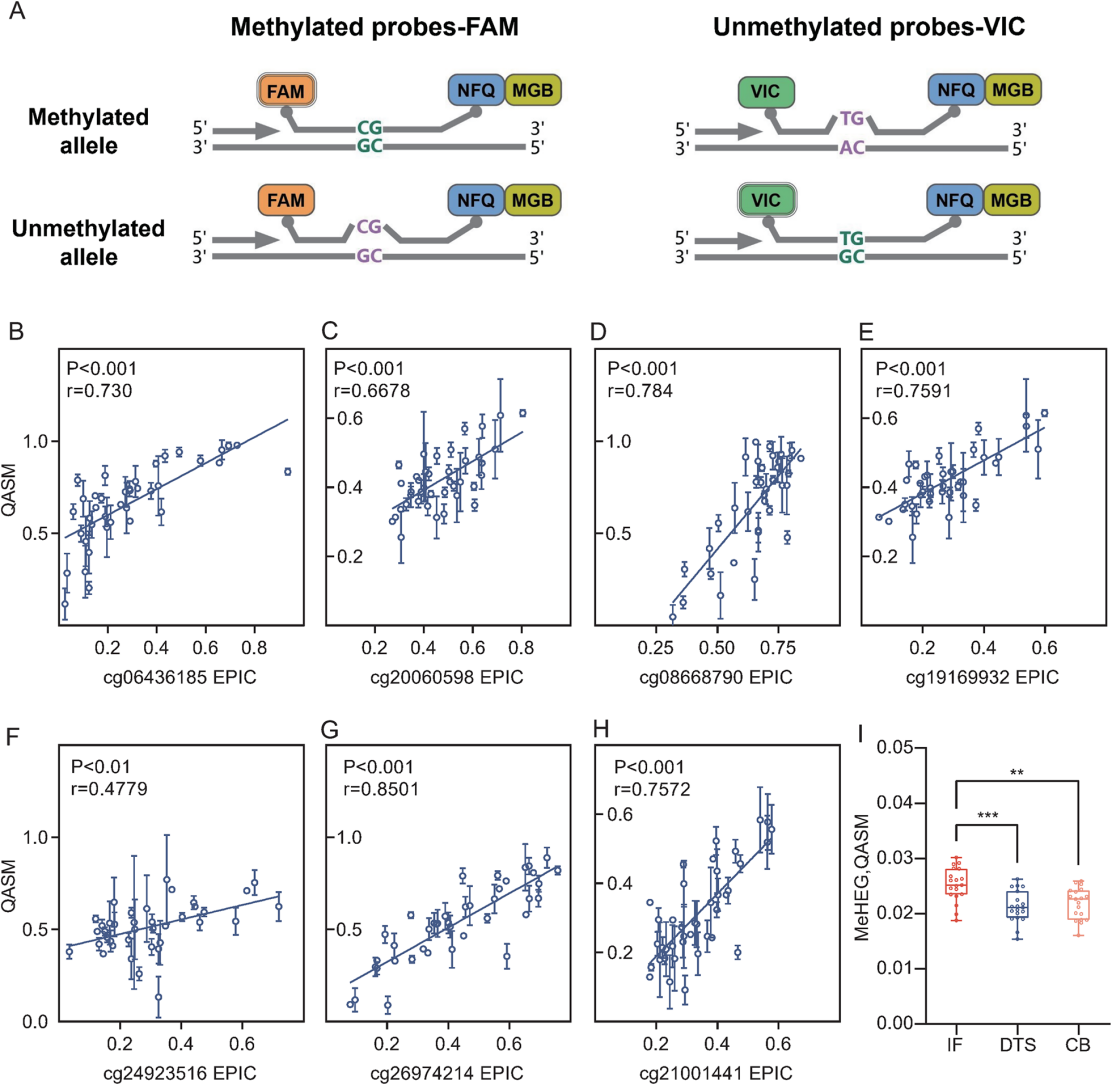
Developing a qPCR-based assay for MeHEG. **A** Concept drawing of the strategy of QASM assay for detection on seven CpG sites included in intratumor DNA methylation heterogeneity. The FAM-labeled probe was specifically bound to sequences derived from methylated allele, and the VIC-labeled probe hybridized to sequences from unmethylated allele. Both alleles were amplified with one pair of primers in the same reaction. **B**-**H** The methylation percentages of cg06436185 (**B**), cg20060598 (**C**), cg08668790 (**D**), cg19169932 (**E**), cg24923516 (**F**), cg26974214 (**G**) and cg21001441 (**H**) determined by QASM assay correlated well with the methylation value measured by EPIC array in 42 samples. Pearson’s test was applied, and the correlation coefficient (r) and p-value for each procedure are shown. **I** MeHEG score determined by QASM assay revealed significant differences among primary intra-tumoral regions (DTS, CB, and IF) in a subset of CRC cases (n = 19). Tukey’s multi-comparison test was applied (***p < 0.001, **p < 0.01)

Among the 7 CpG sites, cg06436185, cg08668790, cg24923516, cg26974214 and cg21001441 were mapped to PRKAG2, ZNF154, CYP27C1, IFIT1 and ATAD3C, respectively. To obtain a MeHEG score based on the above seven probes in a more convenient approach, we developed a qPCR-based assay (quantitative analysis of DNA methylation at single-base resolution, QASM) to determine the methylation level of each CpG site even when it was in a low CpG-density region at a single-base resolution [48]. As illustrated in Fig. 3a, the methylated and unmethylated alleles of each CpG were quantified at the probe level, in which the signal ratio of two probes labeled with fluorescent dyes can determine methylation percentages. To validate the practicality of this qPCR-based assay for determining MeHEG score, we next compared the results of the QASM assay with those of EPIC array in our in-house EPIC cohort. The qPCR-based and EPIC array-based methylation percentages were linearly well-correlated in each CpG site (Fig. 3b-h).

Next, we further confirmed the feasibility of eITH evaluation by MeHEG score determined by QASM assay. Multiple sampling from one tumor is a powerful approach to profiling spatial heterogeneity and inferring tumor populations based on their spatial distributions. To evaluate the eITH within individual tumors, we calculated the MeHEG score by the QASM assay of the IF, CB, and DTS regions, obtained from 19 tumor samples using the laser microdissection. We found that these selected CpGs showed a significant difference in methylation levels among the three regions (Supplemental Fig. S1A-G). The most epigenetically divergent region was the IF, generally known as the crucial interface for molecular changes in CRC. The MeHEG score consistently displayed remarkably different levels among each region within one tumor (Fig. 3i). In contrast, the IF region exhibited the highest eITH according to the MeHEG score (Fig. 3i), thus supporting the qPCR-based MeHEG score as an accurate surrogate for the array-based approach to representing the eITH in CRC. Altogether, these results confirmed the idea that eITH could be assessed by the different region MeHEG score within individual tumor, which could be determined by an efficient and convenient method—QASM assay.

### Epigenetic heterogeneity contributes to shaping the immune landscape in tumor microenvironment

**Fig. 4:**
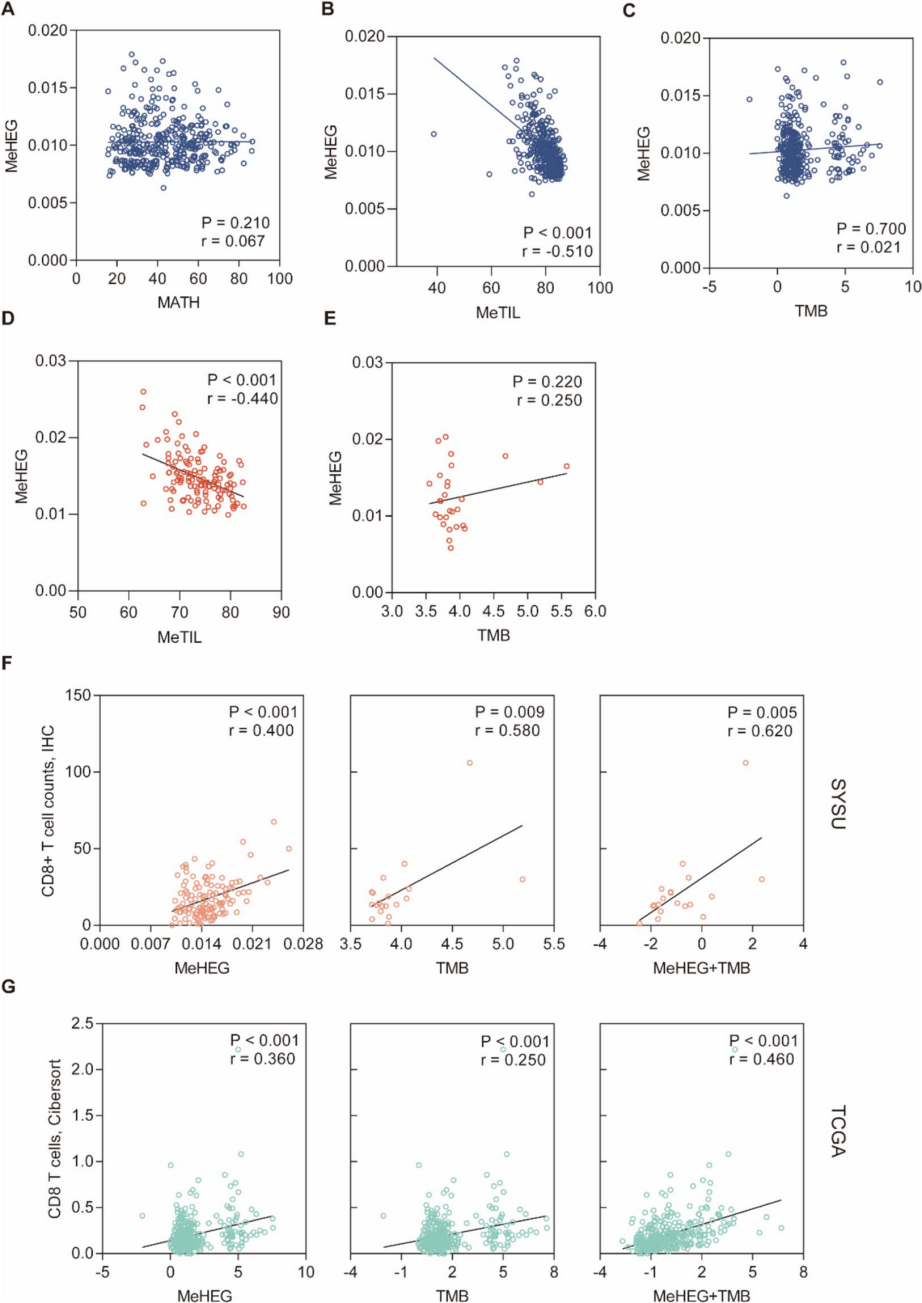
MeHEG-based eITH correlated with tumor immune landscape. **A**-**C** Scatter plots showed the correlation between MeHEG score and MATH score (**A**), MeTIL score (**B**), TMB (**C**) respectively in the TCGA cohort. **D**-**E** Scatter plots showed the correlation between MeHEG score and MeTIL score (**D**), TMB (**E**) respectively in the NEPDC cohort, implying the accumulation of alterations in methylome of the tumor was an independent event from its counterparts and higher eITH might be accompanied by denser CD8 T cells infiltration. **F**-**G** Scatter plots showed the correlation between the density of CD8+ TILs infiltration and MeHEG, TMB alone or combined in NEPDC (**F**) and TCGA (**G**) cohorts, respectively. The CD8+ TILs infiltration were assessed by IHC or CIBERSORTx. The MeHEG and TMB were both positively correlated with denser CD8+ TILs, and could better predict the CD8+ TILs when combing together. Pearson’s test was applied, and the correlation coefficient (r) and p-value for each procedure are shown. *MATH* Mutant-alle Tumor Heterogeneity, *TILs* tumor-infiltrating lymphocytes, *TMB* tumor mutation burden, *IHC* immunohistochemical assay

Apart from epigenetic changes, intratumoral heterogeneity is driven by genetic alternations and tumor microenvironment (TME), so we further investigated their association. To infer intratumoral genetic heterogeneity (gITH), we applied a reference-based mutant-allele tumor heterogeneity (MATH) score based on the mutant-allele fractions derived from WES data. By calculating the MATH score and MeHEG score in the TCGA COREAD cohort, we found no significant correlation between the gITH and the eITH (Fig. 4a), which is consistent with the previous consensus that the process of the intratumoral epigenetic evolution is independent of that of the gITH.

As one major component of the TME, the tumor-infiltrating immune signature was well reported to be involved in tumor evolution. Hence, we performed eITH together with the MeTIL algorithm in our in-house cohort to assess the intratumoral immune infiltrates from histopathological and high-throughput perspectives. The MeTIL algorithm is a methylation-based approach that was developed in our previous study for evaluation of tumor-infiltrating immune cells. Our results showed a negative correlation between MeHEG and MeTIL scores (Fig. 4b, d). Besides, we calculated MeTIL score in HGTP-BHVD cohort and found a significant MeTIL score difference among three regions (Supplemental Fig. 2a), and Tukey’s test showed the IF had the lowest MeTIL score compared with CB and DTS (*P* < 0.001, respectively) (Supplemental Fig. 2a). In HGTP-BHVD cohort, MeHEG score was negatively correlated with MeTIL score in all combined samples (Supplemental Fig. 2b) and three different regions (Supplemental Fig. 2c-e). We further investigated the association between the eITH and the tumor mutation burden (TMB), which was considered to reflect a panoramic tumor mutation landscape, and correlate with the density of immune infiltrates. Similarly to gITH, we found TMB was not associated with the eITH (Fig. 4c, e). We found that the eITH inferred by MeHEG was significantly correlated with the CD8 tumor-infiltrating lymphocytes (TILs) yielded by the IHC (Fig. 4f). MeHEG scores were positively correlated with CD8 + T cell infiltration in tumor tissue (Fig. 4f). On the contrary, MeTIL scores were negatively correlated with CD8 + T cell infiltration in tumor tissue (Supplemental Fig. 2f). Notably, when directly combining the normalized TMB and the MeHEG score, the new linear regression model had a better correlation coefficient than TMB and MeHEG alone (Fig. 4f). The F-test showed that the multiple linear regression model was better than the simple model with any covariate alone.

Similar procedures were applied in the TCGA cohort as an external validation. Given no available histopathological TILs data for the TCGA cohort, we applied an algorithm based on the deconvolution of bulk tumoral mRNA sequencing data, the CIBERSORTx, and the MeTIL algorithm together to infer the TILs. CIBERSORTx is believed to represent the total level of CD8 + T cells in the bulk tumor samples. The infiltration level inferred by CIBERSORTx was considered reliable and as a standard when studying the immune cells infiltration in TCGA samples [49]. Expectedly, consistent results were found in the COREAD cohort (Fig. 4g, Supplemental Fig. 2g). Contributed by a larger number of patients, the F-test of the multiple linear regression model confirmed a significant correlation coefficient change in the model with the TMB and MeHEG together. Taken together, these findings suggest that the TMB and the MeHEG are two independent but complementary tumoral characteristics, and together they provide a more comprehensive perspective on tumor-infiltrating immune signature.

### Epigenetic heterogeneity provides a new perspective in clinical settings

**Fig. 5:**
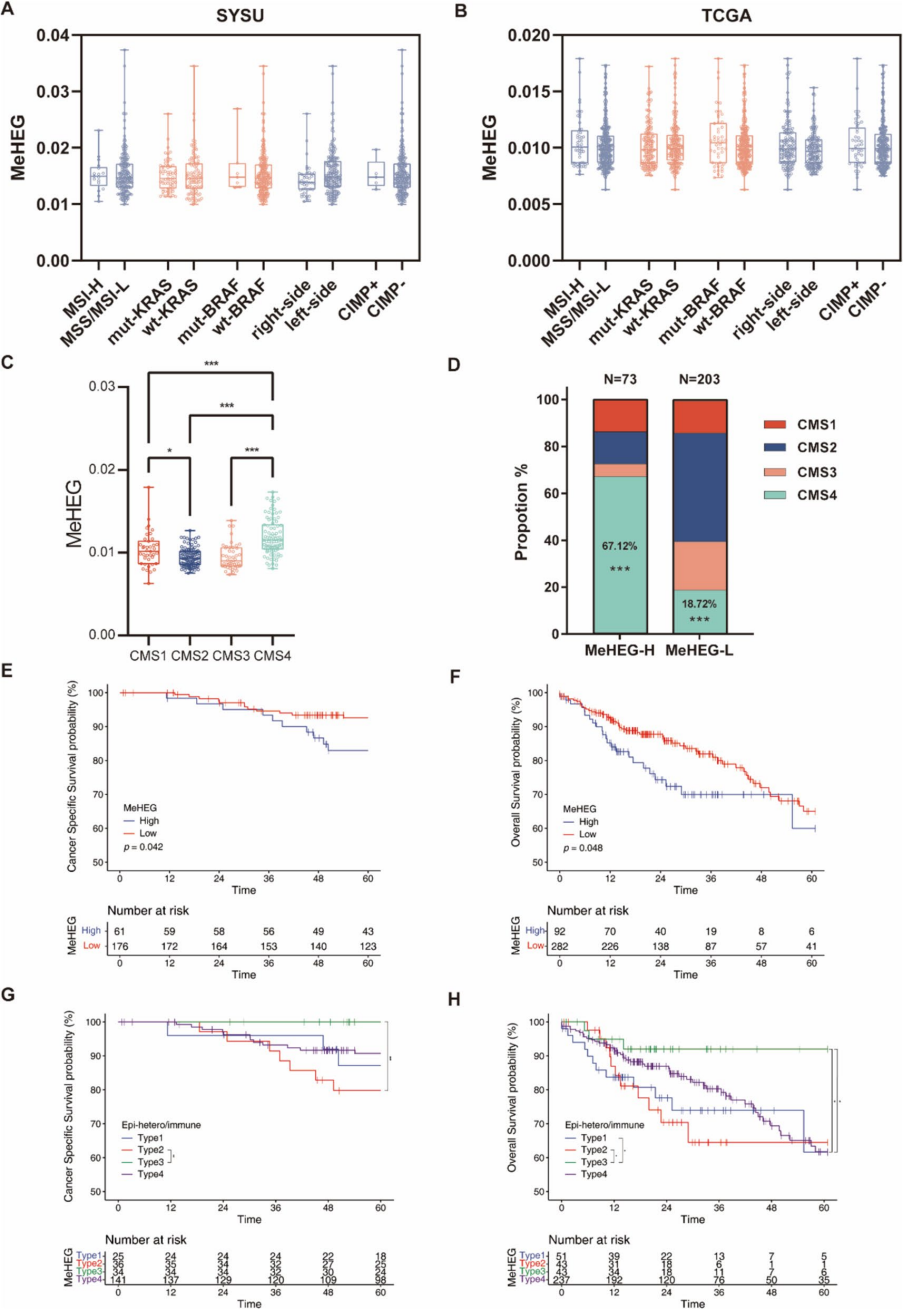
Application of MeHEG-based eITH in clinical settings. **A**-**B** Distribution of the MeHEG score by different molecular phenotypes in the NEPDC cohort (**A**) and TCGA cohort (**B**) respectively. Horizontal bars, min to max. **C** The MeHEG score in four CMS types in the TCGA cohort. **D** The distribution of CMS types in the MeHEG-H and MeHEG-L groups based on a cut-off value of upper 75% quantile. The significant distribution difference could be found in CMS4 types, as the CMS4 was accompanied by a higher MeHEG score. Tukey’s multi-comparison test and Fisher’s exact test were applied (***p < 0.001, **p < 0.01, *p < 0.05). **E**–**F** The prognostic value of MeHEG score in the TCGA cohort. The patients were classified as MeHEG-H and MeHEG-L based on a cut-off value of upper 75% quantile. Kaplan–Meier curves of cancer specific survival (CSS) (**E**) and overall survival (OS) (**F**) with p values from the log-rank test are shown. (G-H) Patients in NEPDC cohort were further stratified by the MeHEG score (same as above) and the MeTIL score (lower 25% quantile was applied as the cut-off value) into four groups (type 1, MeHEG-H/MeTIL-L; type 2, MeHEG-H/MeTIL-H; type 3, MeHEG-L/MeTIL-L; type 4, MeHEG-L/MeTIL-H). Kaplan–Meier curves of CSS (**G**) and OS (**H**) in each cohort are shown. A significantly better prognosis was observed in type 3, while the best prognosis was observed in type 2. (***p < 0.001, **p < 0.01, *p < 0.05)

Considering the application of the gITH in clinical settings, we sought to evaluate the role of the eITH by the MeHEG in its association with clinical characteristics and prognosis. Both in the in-house and the TCGA cohort, the level of the MeHEG score had no significant difference among several different clinical statuses, including the location of the tumor, KRAS/BRAF mutation status, and CpG island methylator phenotype (CIMP) status (Fig. 5a, b). The MSI-H patients showed a higher MeHEG score tendency than MSS/MSI-L patients, though the difference is insignificant (Fig. 5a, b). Notably, we found those with the Consensus Molecular Subtypes (CMS) 4 tumors, featured by harboring a stromal infiltration behavior and worse outcomes [50, 51], had the highest MeHEG score (Fig. 5c), consistent with the finding that the IF region also exhibits the highest MeHEG score. In addition, we divided patients into MeHEG-high and MeHEG-low subgroups by using the upper quartile as the cut-off value. The MeHEG-high group had a significantly higher proportion of the CMS4 tumors compared with the MeHEG-low group (67.12% vs. 18.72%, *P* < 0.001; Fig. 5d). Consistently, the survival outcomes of the MeHEG-high group in both two cohorts were worse than that in the MeHEG-low group, implying tumors with high eITH may possess a more aggressive feature (Fig. 5e, f). Poorer prognosis in MeHEG-high group was consistent with the dismal prognosis in CMS4 tumor.

We further combined two aforementioned probe-based approaches, the MeHEG and the MeTIL, to provide a comprehensive heterogeneity-immune landscape of CRC tumors. By adopting the lower 25th quantile of the MeTIL as the cut-off value, the MeHEG-high and -low groups were further classified as four types (type 1, MeHEG-H/MeTIL-L; type 2, MeHEG-H/MeTIL-H; type 3, MeHEG-L/MeTIL-L; type 4, MeHEG-L/MeTIL-H). We found that the type 3 patients, who harbored lower eITH and higher density of CD8 infiltration, had the best prognosis. Conversely, the type 2 patients, characterized by its higher eITH and lower immune infiltrates, had the worst survival outcomes, significantly worse than that of type 3 in both cohorts (Fig. 5g, h). Notably, although relative to the 450k array-based score in the TCGA cohort, the score of the in-house cohort was calculated based on the QASM, the reproducible results further verified the robustness and practicality of the QASM-based approach.

### Assessment of MeHEG-based eITH in cancer

**Fig. 6:**
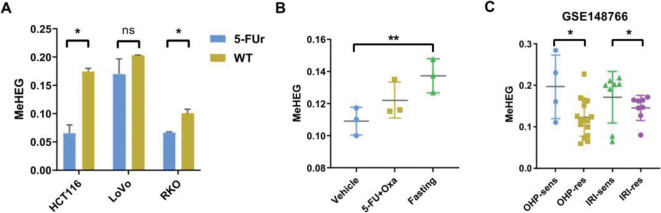
Application of MeHEG in predicting treatment outcome and response to stimulus. **A** MeHEG scores of HCT116. LoVo and RKO wild type cells and their corresponding 5-FUr cells were assessed via QASM assay. **B** MeHEG scores of RKO xenograft after treated with 5-FU and Oxaliplatin or fasting. **C** QASM assay detected MeHEG scores of samples from GSE148766. *5-FUr* 5-FU-resistant CRC cells. (**p < 0.01, *p < 0.05)

Next, we sought to trace the dynamic change of epigenetic heterogeneity in cancer cells under the exerted stress and treatment of therapeutic drugs. Epigenetic remodeling is responsible for tumor progression and drug resistance in CRC. The invariable emergence of acquired drug resistance not only limits the duration of tumor response but also represents the major obstacle for a more meaningful impact on long-term survival in genotype-matched precision medicine. Considering the contribution to therapy resistance of ITH, we established 5-fluorouracil (5-FU) resistant CRC cell lines to investigate the possible epigenetic heterogeneity changes after receiving 5-FU treatment (Fig. 6a). Results showed that CRC cells with resistance to 5-FU presented lower MeHEG level than the wild-typed groups (Fig. 6a). Besides, we calculated the MeHEG scores of RKO xenograft in mice after treated with 5-FU and Oxaliplatin or fasting and found drug treatment could increase eITH (Fig. 6b). More intriguingly, fasting resulted in the highest MeHEG score among those three groups (Fig. 6b). To investigate whether epigenetic heterogeneity would be triggered while receiving treatment, we chose several first-line drugs and targeted drugs used in clinical therapy for in vitro analysis on the eITH levels of CRC cell lines. In vitro drug response of CRC cell lines was heterogeneous and depended on their MeHEG levels. To strengthen our results, we retrieved several datasets from NCBI Gene Expression Omnibus repository which provided methylation profilesand clinical information. The 24 CRC patients that were primarily resistant to first-line drugs (oxaliplatin and irinotecan) were compared with the 12 drug-sensitive CRC patients in GSE148766 dataset [52], and MeHEG score was found to be decreased in the resistant groups (Fig. 6c).

## Discussion

As one component of the ITH, the eITH alterations has long been discussed to be involved in tumor progression and metastasis [22, 53]. The invasive front of a tumor is typically considered an evolutionarily newer part, while the central bulk or digestive tract surface is regarded as the older, more established region of the tumor [54]. By examining multiple anatomic regions within a tumor, we are able to infer the intratumoral epigenetic heterogeneity [55]. However, the current approaches for assessing the epigenetic heterogeneity rely on the complex sequencing manner, regardless of the multi-sampling or single-cell methods, thus limiting further investigation and fast application in research and clinical settings. Besides, several groups have developed computational methods to infer the heterogeneity of cell composition from DNA methylation profiles derived from bulk samples [56, 57]. Nevertheless, no direct methods are currently available for computing the eITH from a bulk tumor sample. Enabling by the fine multiregional sampling by the original studies, which contributed to the high consistency of components among each sample, we acquired the most variant CpG probes among the different regions. By applying the ν-SVR method [58, 59], a novel machine learning method, to deconvolve the methylation mixture profile, we further reduced the dimension of the probes and subsequently constructed the MeHEG algorithm, which can be leveraged to impute the eITH from a bulk profile.

Among the seven finally yielded probes through the ν-SVR, five were mapped to certain genes, leaving two probes in the low-CpG density region. Previous studies mostly accessed these isolated CpGs through the methylation array or pyrosequencing. However, the novel QASM assay developed by our group enables the determination of the single-CpG methylation in a qPCR-based manner and has been validated in independent groups [48, 60]. Indeed, this approach was validated in the current study by the highly consistent results between the QASM and the EPIC array. Facilitating the QASM, we further developed a sequencing-free approach to yield the MeHEG score. Through the microdissection and multi-sampling, we measured the eITH, represented by the MeHEG score, finding that the IF region had the highest eITH, which agreed with the consensus that the IF acts as the interface of cancer and the immune context, and thus the residents in IF have been undergoing the highest selective pressure [53, 61].

The ITH arises from the accumulation of spontaneous mutation or alteration in genome and epigenome and forms the regional pattern by the selective pressures from the distinct TME [61]. Though shared an agreement in the evolutionary histories, the association between the genetic and methylation variability might not attribute to causality [62]. Furthermore, previous studies have shown that the eITH and gITH might contribute independently to the tumorigenesis and progression [43, 63]. Herein, our results also suggested the independence of eITH from its counterparts and, moreover, indicated that the degree of eITH was also not directly associated with TMB. By evaluating the immune infiltrates from the pathological and deconvolutional methods, we further revealed that higher eITH, inferred by the MeHEG score, was well correlated with denser CD8 TILs. From the other perspective, IF region had the highest eITH, which means the region is filled with CD8 TILs, which agree with the consensus that the IF acts as main battlefield between cancer and the  immune system. This association between eITH and immune response seemed to be different from that of gITH, as the robust immune response could limit the variety of tumoral subclones, and a higher gITH was corresponding to a diminished immune activation [64, 65]. Since the epigenome is highly plastic relative to the genome, cancer cells can display a rapid epigenetic evolvement under the selective pressures from the TME [29, 66]. Thus, the eITH and the immune infiltrate may have a complex reciprocal cause and effect relationship. Further study is needed to fully elucidate this relationship, which could provide new insights for tumor immunotherapy.

Regarding contributions to the phenotypes, the eITH has been suggested by many studies to be associated with certain tumoral behaviors, including immune escape, metastasis and drug response [22, 55, 67]. However, this study found no difference in the eITH between mismatch repair (MMR) status, CIMP status, and the BRAF/KRAS mutation phenotype in the in-house and the TCGA cohorts. In contrast, we identified that the CMS4 tumor, the mesenchymal type, which is characterized by stromal infiltration and angiogenesis [51], had a significantly higher eITH. CMS4 tumor shows dismal prognosis, which is consistent with worse survival outcomes in tumors with high eITH. Besides, CMS4 tumor, with high eITH, behaves more EMT phenotype. Cancer cells in IF region, also with high eITH, become more aggressive through EMT. Therefore, CMS4 tumor have higher eITH might due to more proportion of IF region within tumor tissue. This finding, together with the difference of the eITH in the three tumoral regions, suggested the notion that increased eITH was associated with a more aggressive phenotype. Likewise, a shorter survival was also identified in patients with increased heterogeneity. We classified patients into four types by combining the MeTIL and the MeHEG. Type 2, which had a higher eITH and lower infiltrates and thus represented the outcomes of “successful” evolution of tumors in immune escape, exhibited the worst survival. Though further validation is needed, this subtype might be the candidates for DNA methylation inhibitors therapies. Of note, the algorithms mentioned above were implemented based on the results of the qPCR in the in-house cohort relative to the array-based ones in the TCGA, therefore suggesting their robustness in both array-based and PCR-based manners, and they could be directly applied in the currently available high throughput data or a fast assessment for desired samples just on the bench.

We used MeHEG algorithm to evaluate the dynamic change of eITH in cancer cells under the exerted stress and treatment of therapeutic drugs. Our results showed that 5-FU-induced resistant CRC cell line showed lower MeHEG score than control group, which indicated that persistent 5-FU resistant cancer cells possessed a lower eITH. Drug treatment killed all the cancer cells except persistent 5-FU resistant cells, the latter was a subset of cells with high homogeneity. Whereas, our xenograft model showed that drug treatment could induce higher eITH compared with control group, which might be due to transient effect of drug-triggered epigenetic change. Interestingly, the fasting group in xenograft mice resulted in higher eITH compared with 5-FU group, which might be explained by the stress response that could trigger an explosive epigenetic alteration. Altogether, drug treatment could boost epigenetic alterations which induce a higher eITH at early stage, but lead to a lower eITH finally when they kill all the subset of cells except the drug-resistant ones. Different duration and concentration of the drug treatment might be the reason why they have the opposite effect. The oxaliplatin- and irinotecan-resistant groups also showed a decreased eITH compared with control group, which was consistent with the notion that persistent drug resistant cancer cells possess lower heterogeneity [52].

There are some shortcomings and limitations in our study. Specifically, the underlying mechanism of the involvement of the methylated CpGs targeted by the seven probes in the tumoral evolution and clonal diversity was not clearly understood. Indeed, due to the “black box” distinct of the learned models, it is difficult to translate the patterns responsible for the output into human-understandable descriptions [68]. Nonetheless, the selected CpGs sites could still provide insights into future investigations. Moreover, larger and multicenter clinical specimens are needed for further validation of MeHEG score-based eITH evaluation.

In conclusion, our study presents the development of a fast and reliable algorithm, MeHEG, for inferring eITH in CRC. MeHEG further enables an array-free assessment based on the QASM methods (Fig. 1). We validated that eITH was independent of genetic variation and observed a positive association between increased eITH and the presence of CD8 + TILs. Using our MeHEG tool, we found that CMS4 tumors exhibited higher eITH and eITH increased under drug and energy stress conditions. Conversely, tumors with drug resistance displayed lower eITH. These findings underscore the potential of MeHEG as a robust tool for assessing epigenetic heterogeneity and its clinical implications in understanding CRC progression and treatment responses.

## Methods

### Study population and samples

The patient samples used in this study were collected from the National Basic Research Program of Evolution from Precancerous Disease to Cancer in China (NEPDC cohort, No. 2015CB554000) [60, 69, 70] and HGTP-BHVD cohort derived from two Spanish medical centers [43]. First, the samples of different tumor regions in the slides of 79 CRC patients from HGTP-BHVD cohort were used to generate spatial-specific methylation profile. Then, we generated a cohort by collecting the fresh-frozen tumors and adjacent normal tissues of 45 CRC patients from NEPDC cohort for methylation array analysis. Next, to validate the findings, we used the formalin-fixed paraffin-embedded (FFPE) tissue of 359 patients from the NEPDC cohort to generate an independent set of tumors. The external validation used the COAD and READ cohorts released by The Cancer Genome Atlas (TCGA) project. Specifically, we included patients with available Illumina human methylation 450 k BeadChip data. The flow for patient disposition in each cohort was summarized in Supplemental Fig. S3. This study was approved by the Institutional Review Board of Sun Yat-sen University. All patients have given written informed consent.

### Analysis of the HGTP-BHVD cohort

In the HGTP-BHVD cohort, each sample included methylation data from three distinct tumor regions. When performing the DMP analysis, we incorporated intertumoral differences by comparing these three regions. This method has been previously employed by the original study [43] to assess intertumoral heterogeneity, where methylation profiles across different tumor regions were compared to highlight the diversity within and between tumors. By addressing intertumoral heterogeneity in this way, we preserved the sensitivity to detect regional variations in epigenetic marks, ensuring that the DMPs we identified were specific to the different tumor regions rather than being overly influenced by intertumoral variability.

### Methylation array analysis

The DNA methylation status of 865,859 CpG sites in each tissue sample was profiled by using the Infinium Methylation EPIC BeadChip (EPIC array). We interrogated genome-wide methylation at single-nucleotide resolution in the EPIC cohort containing 45 fresh-frozen tumor samples from the NEPDC using this array following the manufacturer’s protocol. The probes data filtering, normalization, and DMP analysis were performed using the “minfi” package with standard protocols [60, 71].

### Deconvolution model

Several different reference-based methods have previously been employed to deconvolve methylation mixture profiles, both with [72, 73] and without [74, 75] unknown content and several reference-free deconvolution methods [57, 76]. Let B denote a DMP signature matrix and let f denote a vector consisting of the unknown fractions of each cell type in the mixture. Then the problem of DMP deconvolution can be represented by m = f × B, provided that B contains more marker genes than content types (i.e., the system is overdetermined4). If the linearity argument is biologically plausible, then CpG methylation profiles enriched in each content type can be leveraged to impute unknown content fractions from mixture profiles.

We proposed a novel approach for content-type estimation by evaluating relative subsets of DNA methylation profiles: MeHEG. We applied the reference-based deconvolution methods. Our strategy is based on a novel application of ν-SVR, an instance of support vector machine, a machine learning method that minimized overfitting and outperformed other approaches in benchmarking experiments as previously demonstrated [58, 59, 77]. Notably, support vectors represent the CpG locus selected from the methylation signature matrix in this work. The nu parameter serves as an upper bound on the training errors and a lower bound on the fraction of support vectors. Our current implementation of MeHEG executes ν-SVR using the “svm” function in the R package e1071. Negative SVR regression coefficients are subsequently set to 0 (as is done for LLSR), and the remaining regression coefficients are normalized to sum to 1, yielding a final vector of estimated content type fractions, f (notably, f denotes relative, not absolute, fractions of each content type from B in m). We calculated the MeHEG score using the coefficient of variation of region-specific weighted beta values for the selected probes. The formula is as follows:

$$MeHEG= \frac{\sqrt{\frac{1}{R}{\sum }_{j=1}^{R}{(\sum_{i=1}^{P} {\beta }_{i,k} \cdot {w}_{i,j}-\overline{S })}^{2}}}{\overline{S} }$$

where: *R* is the number of regions. *P* is the number of probes. *β*_*i,k*_ is the Beta value for probe* i* in sample *k*. *w*_*i,j*_ is the weight of probe *i* for region *j*. $\overline{S }$ is the mean of the regional scores for a sample across all regions. The MeHEG score thus represents the variability within a tumor. A higher MeHEG score indicates greater eITH. Moreover, we developed an algorithm and corresponding R package MeHEG to facilitate calculations.

### Laser microdissection

Tumor tissues used for this study came from the Sixth Affiliated Hospital of Sun Yat-sen University. All samples corresponded to FFPE sections archived at the Department of Pathology. The gastrointestinal pathologist selected 20 CRC tissues from patients with rich tumor cell populations (at least 80%) using an optic microscope, consecutively diagnosed and treated with surgical resection from 2019 to 2020. All the picked tissues were stained with hematoxylin and eosin and subsequently defined and microdissected into three regions with ZEISS PALM MicroBeam: DTS, CB and IF. Colored labels illustrate multi-region sampling of each colorectal cancer. FFPE sections should be routinely deparaffinated using xylene and ethanol because paraffin would reduce laser efficiency. After deparaffination, we performed H&E staining for subsequent observation and isolation. The collective tube was put into the collector in an upside-down position with capturing buffer in the cap. Check the right position of the correction collar. After defining three tumor regions, we used a focused laser beam to precisely cut out and isolate selected specimens. Then the laser catapult collected the target area into sterilized microfuge tubes, which allowed us to obtain exactly separated specimens without contamination. We centrifuged the collective tube at full speed for 5 min and performed a DNA extraction procedure.

### DNA extraction and bisulfite conversion

For FFPE sections and microdissected samples, we used DNA FFPE Tissue Kit (QIAGEN, Hilden, Germany) following the recommended protocol. According to the manufacturer’s instructions, we used the DNeasy Blood & Tissue Kit (QIAGEN, Hilden, Germany). DNA was quantified using NanoDrop ND-2000 spectrophotometer (NanoDrop Technologies, DE, USA). Purified DNA was denatured and modified with sodium bisulfite using EZ DNA Methylation Kit (Zymo Research, CA, USA), where cytosine was converted into uracil.

### qPCR-based methylation profiling at single-base resolution

Bisulfite-treated DNA was used as a template for QASM using specific primers for the target CpG sites. We have developed this technique and showed its robustness in methylation profiling at single-base resolution in the previous studies [48, 60, 70]. Primer and probe sequences used in the QASM assay are listed in Supplemental Table S3. The reaction mixture of 20 μl contained PCR Buffer, dNTPs, primers, FAM/NED-labeled probes, VIC/NED probes, bisulfite-converted DNA and HotStarTaq DNA polymerase (QIAGEN). Fluorescence-based real-time PCR assays were run in duplicate with the Applied Biosystems^®^ QuantStudio 7 Flex Real-Time PCR System (Thermo Fisher Scientific, MA, USA). We calculated the methylation percentage of specific CpG sites of each sample as follows: Methylation Percentage = 100/(1 + 1/2-△CT), △CT = CT methylation—CT unmethylation.

### Molecular characterization for tumor

The microsatellite stability and instability (MSS/MSI-low/MSI-high) was assessed based on immunohistochemistry testing of MLH1, MSH2, MSH6, and PMS2 [78, 79]. The hotspot mutations in *BRAF* p.V600E and *KRAS* codons 12 and 13 were identified through Sanger sequencing [80]. CpG island methylator phenotype (CIMP) was determined by the quantitative methylation-specific PCR assay using the Weisenberger’s panel (*CACNA1G*, *IGF2*, *NEUROG1*, *RUNX3*, *SOCS1*) as previously described [81, 82].

### Assessment of tumor-infiltrating lymphocytes

TILs were assessed with histochemical assay based on the standard protocol described previously [60, 83]. Briefly, the FFPE tumor blocks were cut into 4 µm sections. The deparaffinized slides were incubated with the monoclonal anti-CD8 antibody (clone C8/144B, DAKO, Kyoto, Japan, SK201) at 1:100 dilution for 16 h at 4 °C. The secondary antibody conjugated with horseradish peroxidase was applied. Two independent pathologists performed visual enumeration in five representative fields (0.1255 mm2 per field), and the mean counts of CD8 + TILs were recorded. In addition, machine learning approaches were also applied to deconvolute the density and types of immune cells. For bulk RNA-seq data, the CIBERSORTx algorithm was applied to calculate the absolute fraction scores of each cell type. For bulk methylation array data, we used the MeTIL algorithm [60] that has been developed and validated in our previous study to infer the density of CD8 + TILs. Briefly, the CD8 + MeTIL was constructed by using CD8 + T cell-specific DMPs that were identified from Illumina EPIC methylation arrays. This DNA methylation-based MeTIL score is a reliable tool to evaluate CD8 + T cells infiltration. Lower MeTIL score represents higher CD8 + T cells density. A corresponding PCR-based assay for quantitative analysis of DNA methylation at single-base resolution is also available to determine MeTIL score in a fully quantitative, accurate, and simple manner.

### Whole-exome sequencing profile

WES was performed in 30 patients from the NEPDC cohort. Briefly, genomic DNA was used for library preparation using the Agilent SureSelect Human All Exon kit V6 (Agilent Technologies, Santa Clara, USA). 500 ng of the enriched library was used in the hybridization and captured. Following hybridization, the captured libraries were purified according to the manufacturer’s recommendations and amplified by polymerase chain reaction. Normalized libraries were pooled, and DNA was sequenced on the Illumina HiSeq 2000 using 2 × 150-bp paired-end reads; an average of 39 million reads were sequenced per tumor sample (average 123 × the mean tumor target coverage). WES data were used to generate tumor mutation burden (total number of nonsynonymous mutations per megabase.) for each patient.

Genetic tumor clonal heterogeneity was quantified using the Mutant-Allele Tumor Heterogeneity score, defined as the median absolute deviation of the mutant-allele fraction divided by the median mutant-allele fraction [84]. The MATH score was calculated with the R package “maftools” based on the Mutation Annotation Format files processed from the WES analysis pipeline. The Variant Call Format files generated by our in-house WES data processing were converted into MAF files using “vcf2maf v1.6.21”. The preprocessed MAF files of the TCGA cohorts were obtained from Broad Institute using the R package “TCGAmutations”.

### Cell lines, cell culture and animal experiments

Human CRC cell lines HCT116, RKO and LoVo were obtained from the American Type Culture Collection (ATCC) and cultured in recommended media supplemented with fetal bovine serum (Gibco, Carlsbad, CA) (10%, v/v) according to ATCC guideline. All the cells were maintained at 37 °C in a humidified atmosphere containing 5% CO2 and confirmed to be free from microbiological contamination and cross-contamination.

Indicated 5-FU-resistant CRC cells were established by treating cells with increasing concentrations (up to 200 μM) of 5-FU for more than 6 months in complete DMEM medium.

For floating culture, 100 mm dishes were coated with 0.7% agarose beforehand. Then, HCT116, RKO, LoVo and SW480 cells were cultured in serum-free advanced media (Gibco) in 100 mm dishes (Corning, NY) at a density of 7 × 10^4^ cells/ml.

For tumor xenograft experiment, male BALB/c nude mice (4–6 weeks old) were purchased from the Vital River. The mice were fed and treated following the guidelines approved by the Sun Yat-sen University Institutional Animal Care and Use Committee. RKO cells (5 × 10^6 ^cells/mouse) mixing with Matrigel (25%, v/v) were subcutaneously seeded into mice. Mice were divided into three group which were treated with vehicle, fasting and 5-FU + Oxaliplatin respectively. Tumor MeHEG score were calculated with QASM assay.

### Statistical analysis

Statistical analysis was performed using SPSS Statistics 25.0 (IBM, USA), GraphPad Prism 9.2.0 (GraphPad Software, USA) and R version 4.0.5 (The R Foundation). The Pearson’s or Spearman’s correlation test was used to analyze the relationship between two variables. Comparison within groups was conducted using Student’s t-test, Mann–Whitney U test, Chi-square test or Fisher’s exact test as appropriate. The performance of multiple linear regression was evaluated by the F test. Survival analysis was performed for overall survival and cancer-specific survival through Kaplan–Meier method. A *P* value < 0.05 was considered statistically significant.

## Supplementary Information

Additional file 1.

## Data Availability

All the methylation array data of NEPDC and HGTP-BHVD cohorts have been deposited in the NCBI Gene Expression Omnibus under the accession numbers of GSE119526 and GSE69550, respectively. The whole exome sequencing data have been submitted at the NCBI SRA Database under the accession number of PRJNA803795. The clinical datasets used and analyzed are available from the corresponding author on reasonable request. The ‘MeHEG’ package with the codes for the algorithms developed and used is available at https://github.com/hyu020/MeHEG. The sample codes for using MeHEG could be found in the supplemental materials. The datasets used and/or analyzed during the current study are available from the corresponding author upon reasonable request.

## Abbreviations

ITH: Intratumoral heterogeneity
eITH: Intratumoral epigenetic heterogeneity
MeHEG: DNA methylation-based heterogeneity
CRC: Colorectal cancer
DMPs: Differentially methylated probes
DTS: Digestive tract surface
CB: Central bulk
IF: Invasive front
ν-SVR: Nu–support vector regression
QASM: Quantitative analysis of DNA methylation at single-base resolution
TME: Tumor microenvironment
gITH: Intratumoral genetic heterogeneity
MATH: Mutant-allele tumor heterogeneity
MeTIL: Methylation-based approach for evaluating tumor-infiltrating lymphocytes
TMB: Tumor mutation burden
CIMP: CpG island methylator phenotype
MSS: Microsatellite stability
MSI: Microsatellite instability
CMS: Consensus Molecular Subtypes
5-FU: 5-Fluorouracil
