# Supplementary material for: Spatial deconvolution from bulk DNA methylation profiles determines intratumoral epigenetic heterogeneity
Source: Cell Biosci. 2025 Jan 23;15:7. doi: 10.1186/s13578-024-01337-y (PMC11756021; doi:10.1186/s13578-024-01337-y)
Supplement: Supplementary file 1 — Additional file 1. [file 13578_2024_1337_MOESM1_ESM.docx]

**Supplemental materials**

Supplemental figure 1. QASM assay to determine selected CpGs methylation levels among the three regions

Supplemental figure 2. MeTIL score correlated with MeHEG score and CD8+ T cell.

Supplemental figure 2. MeTIL score correlated with MeHEG score and CD8+ T cell.

Supplemental Table S1. GO analysis of genes that were targeted by intratumor region-specific differentiated methylated probes.

Supplemental Table S2. Intratumor region-specific differentiated methylated probes.

Supplemental Table S3 Oligos used in the current study.


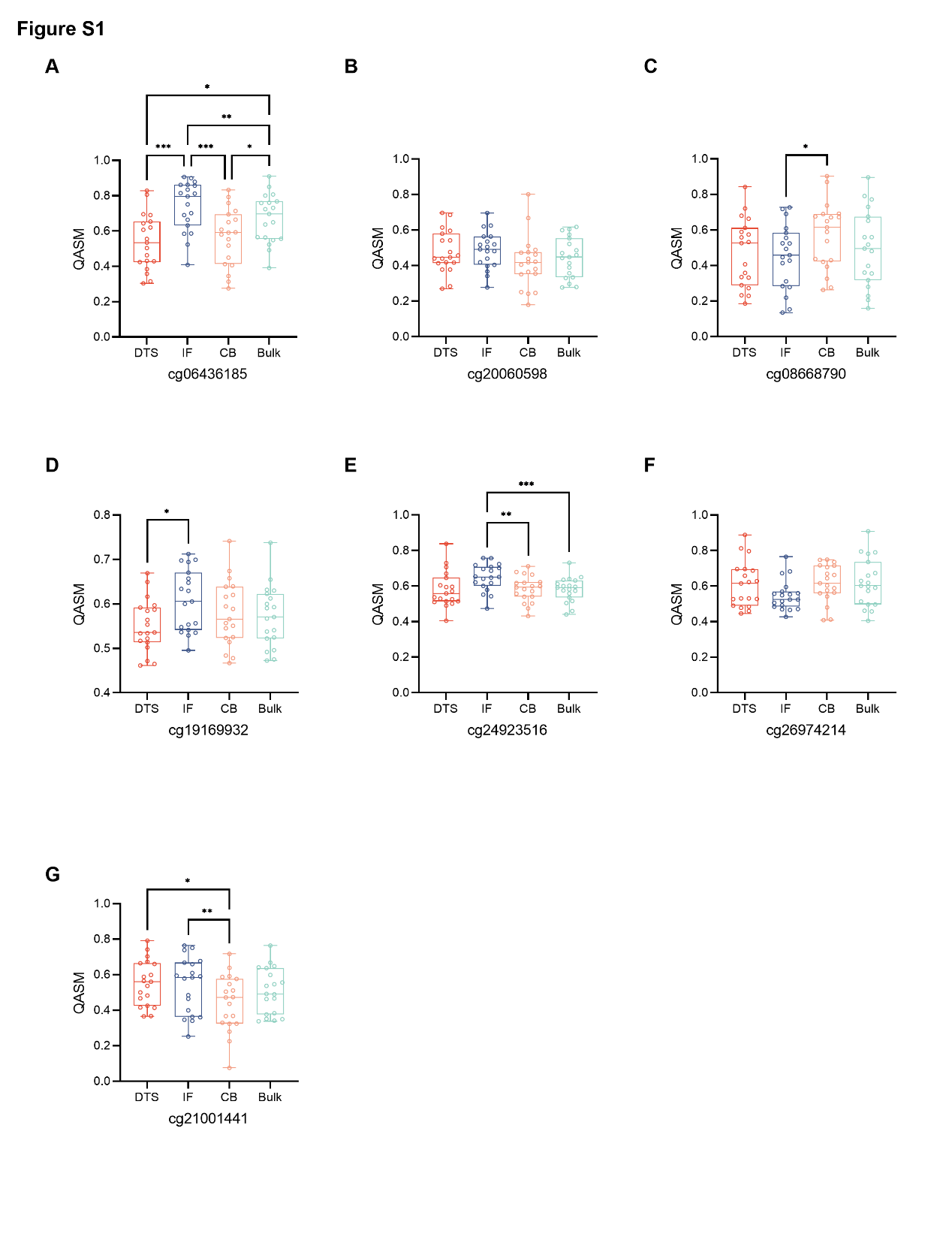


**Supplemental figure 1. QASM assay to determine selected CpGs methylation levels among the three regions**

(A-G) The methylation percentages of cg06436185 (A), cg20060598 (B), cg08668790 (C), cg19169932 (D), cg24923516 (E), cg26974214 (F) cg21001441 (G) determined by QASM assay revealed distinct distributional pattern and significant differences among primary intra-tumoral regions (DTS, CB, and IF) in a set of CRC cases (n=19).


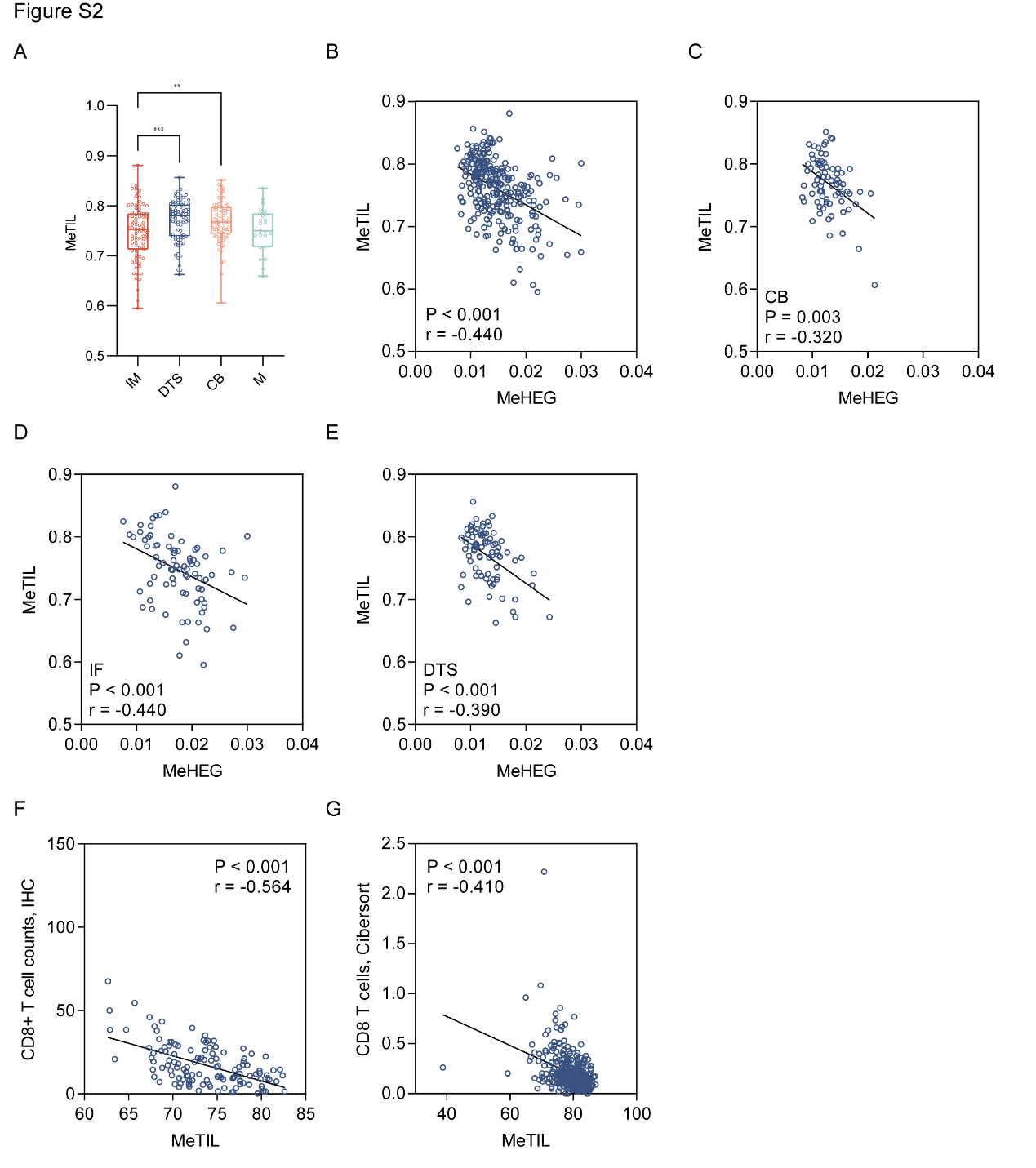


**Supplemental figure 2. MeTIL score correlated with MeHEG score and CD8+ T cell.**

(A) Box plot of the MeTIL score in different primary tumor regions and metastasis. A lower MeTIL score indicated a higher density of CD8 TILs. Tukey’s multi-comparison test was applied (***p<0.001, **p<0.01). (B-E) The scatter plot showed the correlation of the MeHEG and the MeTIL score calculated in all tumor samples (B), DTS (C), IF (D), and CB (E). Pearson’s test was applied, and the correlation coefficient (r) and p-value for each procedure are shown. The correlation in IF is the strongest one among the three regions. (F-G) The scatter plot showed the correlation of the MeTIL score and the CD8 TILs in the SYSU cohort (F) and the TCGA cohort (G), which is evaluated by IHC and CibiersortX, respectively. Aberrative: IF, invasive front; DTS, digestive tract surface; CB, central bulk; IHC, immunohistochemical; SYSU: Sixth Affiliated Hospital of Sun Yat-sen University.


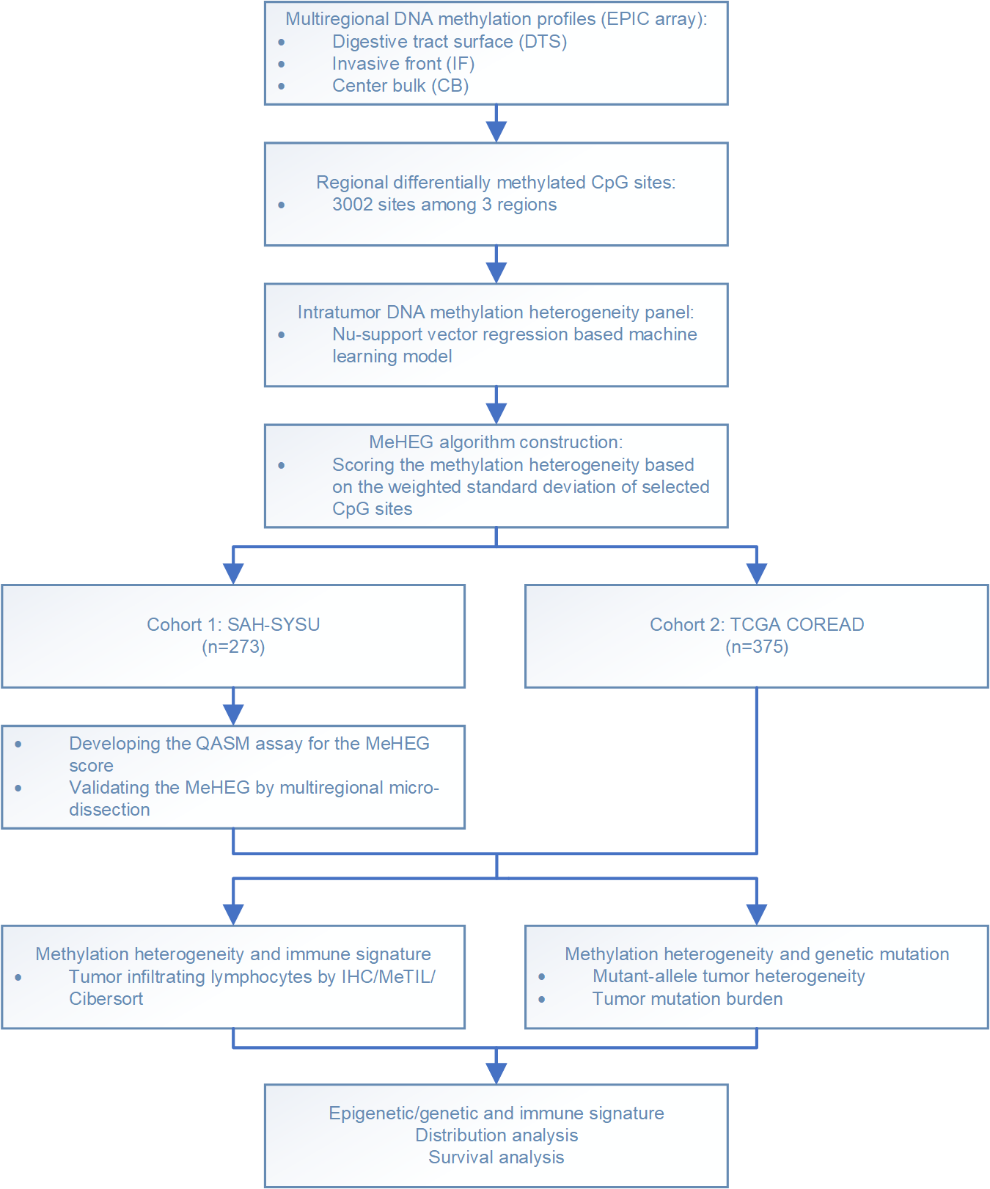


**Supplemental figure 3. Flow diagram of the study.**

Abbreviation: IF, invasive front; DTS, digestive tract surface; CB, central bulk; QASM, Quantitative analysis of DNA methylation: SAH-SYSU: Sixth Affiliated Hospital of Sun Yat-sen University.

**Supplemental Table S1. GO analysis of genes that were targeted by intratumor region-specific differentiated methylated probes.**

|  | **Term** | **-log_10_(*p*-value)** | |
| --- | --- | --- | --- |
| **Biological Process** | localization | | 13.81528199 |
|  | cell differentiation | | 13.51451328 |
|  | cell adhesion | | 13.00535154 |
|  | regulation of localization | | 12.35874202 |
|  | cellular response to stimulus | | 9.74414261 |
|  | extracellular matrix organization | | 9.03930876 |
|  | cell migration | | 8.38577312 |
|  | epithelial cell differentiation | | 8.31299610 |
| **Cell Component** | plasma membrane part | | 20.5187507091 |
|  | cell periphery | | 17.6501838533 |
|  | plasma membrane | | 17.1973866195 |
|  | cell junction | | 16.8283682273 |
|  | plasma membrane region | | 15.8567723465 |
|  | cell-cell junction | | 10.6729062503 |
|  | anchoring junction | | 9.4899765011 |
|  | adherens junction | | 8.9723902037 |
| **Molecular Function** | DNA-binding transcription activator activity, RNA polymerase II-specific | | 7.1277536410 |
|  | cell adhesion molecule binding | | 5.9547027903 |
|  | RNA polymerase II regulatory region sequence-specific DNA binding | | 5.1155779937 |
|  | RNA polymerase II regulatory region DNA binding | | 5.0661810620 |
|  | RNA polymerase II proximal promoter sequence-specific DNA binding | | 4.4059202171 |
|  | cell-cell adhesion mediator activity | | 4.3619200910 |
|  | proximal promoter sequence-specific DNA binding | | 4.1735425695 |
|  | sequence-specific double-stranded DNA binding | | 4.0970908862 |

**Supplemental Table S2. Intratumor region-specific differentiated methylated probes.**

| **rank.MDA** | **Target ID** | **Mean Decrease Accuracy** | **Mean Decrease Gini** | **UCSC Reference Name** | **UCSC Reference Group** | **Relation to CpG island** |
| --- | --- | --- | --- | --- | --- | --- |
| 1 | cg06436185 | 2.5130805 | 0.275918 | PRKAG2 | Body |  |
| 2 | cg20060598 | 2.4626494 | 0.0509613 |  |  | N_Shore |
| 3 | cg08668790 | 2.4448526 | 0.1066075 | ZNF154 | TSS200 | S_Shore |
| 4 | cg19169932 | 2.384332 | 0.1196043 |  |  |  |
| 5 | cg24923516 | 2.3778689 | 0.1720397 | CYP27C1 | Body |  |
| 6 | cg26974214 | 2.33397591 | 0.04369231 | IFIT1 | TSS1500 |  |
| 7 | cg21001441 | 2.29651156 | 0.07146177 | ATAD3C | TSS1500 |  |

**Supplemental Table S3 Oligos used in the current study.**

| **Primers and Probes** | **Sequence (5'-3')** |
| --- | --- |
| PRKAG2 F | GTAGGAGAGTTTTGGTAAATATGTGTTAAGG |
| PRKAG2 R | TTCCAAACCACATCTCATTATACAAAA |
| PRKAG2 FAM | CCCTCCGCGCCGAA |
| PRKAG2 VIC | ATCCCCTCCACACCAAA |
| cg20060598 F | GAGAGGGGGTGATAATTTGATTTT |
| cg20060598 R | AAACAAAACAACCCCACTACAAATAC |
| cg20060598 FAM | GAAGTTGGCGTTGTTAGG |
| cg20060598 VIC | GAAGTTGGTGTTGTTAGGA |
| ZNF154 F | GGGATTTATGAAAATTATATTATTTAGAATGTTTTG |
| ZNF154 R | TCCTTTCTTTTTATAACTCTCAAAAAAAATC |
| ZNF154 FAM | TTAATAACGTAACGTTCAAC |
| ZNF154 VIC | CTCTTAATAACATAACATTCAAC |
| cg19169932 F | GTTGTGGAAAATAGAAGTTGGGTAGTA |
| cg19169932 R | TATTTATAAAACTACTCTAATAACTAAAATTCAACC |
| cg19169932 FAM | GTGTAGGATAGGTACGGTTG |
| cg19169932 VIC | GTGTAGGATAGGTATGGTTG |
| CYP27C1 F | GGGTGGAAAGAGAGAATTAAGATTG |
| CYP27C1 R | CCACCTATTAATAAAATCAATAACTCCTACC |
| CYP27C1 FAM | AAACAAAATATACGTACAATACT |
| CYP27C1 VIC | CAAACAAAATATACATACAATACT |
| IFIT1 F | ATAAGGTTTTTGGTTGTTGGTTGTTT |
| IFIT1 R | CTCTTTCCTTCATTCCTAAACCAAATT |
| IFIT1 FAM | TTTGGTTTTAGTTTGACGGAA |
| IFIT1 VIC | TTTGGTTTTAGTTTGATGGAA |
| ATAD3C F | GATATTTATTTAAAGATAGTTTTTTTTAGGTGATT |
| ATAD3C R | AATTTTTCCTATTAATTACTAATTACCCATTATATC |
| ATAD3C FAM | TTCCTCGAATCACCTAAA |
| ATAD3C VIC | TTTTCCTCAAATCACCTAAA |
